# Circulating peroxiredoxin 4 and type 2 diabetes risk: the Prevention of Renal and Vascular Endstage Disease (PREVEND) study

**DOI:** 10.1007/s00125-014-3278-9

**Published:** 2014-06-04

**Authors:** Ali Abbasi, Eva Corpeleijn, Ron T. Gansevoort, Rijk O. B. Gans, Joachim Struck, Janin Schulte, Hans L. Hillege, Pim van der Harst, Ronald P. Stolk, Gerjan Navis, Stephan J. L. Bakker

**Affiliations:** 1 Department of Epidemiology, University of Groningen, University Medical Center Groningen, Groningen, the Netherlands; 2 Department of Internal Medicine, University of Groningen, University Medical Center Groningen, Groningen, the Netherlands; 3 MRC Epidemiology Unit, University of Cambridge School of Clinical Medicine, Institute of Metabolic Science, Cambridge Biomedical Campus, Addenbrooke’s Hospital, P.O. Box 285, Cambridge, CB2 0QQ UK; 4 AdrenoMed AG, Hennigsdorf, Germany; 5 Department of Research, BRAHMS GmbH (part of Thermo Fisher Scientific), Hennigsdorf, Germany; 6 Department of Cardiology, University of Groningen, University Medical Center Groningen, Groningen, the Netherlands; 7 Department of Genetics, Groningen, University of Groningen, University Medical Center Groningen, Groningen, the Netherlands; 8 Durrer Center for Cardiogenetic Research, ICIN-Netherlands Heart Institute, Utrecht, the Netherlands

**Keywords:** Epidemiology, Peroxiredoxin 4, Risk prediction, Sex difference, Type 2 diabetes

## Abstract

**Aims/hypothesis:**

Oxidative stress plays a key role in the development of type 2 diabetes mellitus. We previously showed that the circulating antioxidant peroxiredoxin 4 (Prx4) is associated with cardiometabolic risk factors. We aimed to evaluate the association of Prx4 with type 2 diabetes risk in the general population.

**Methods:**

We analysed data on 7,972 individuals from the Prevention of Renal and Vascular End-stage Disease (PREVEND) study (49% men, aged 28–75 years) with no diabetes at baseline. Logistic regression models adjusted for age, sex, smoking, waist circumference, hypertension and family history of diabetes were used to estimate the ORs for type 2 diabetes.

**Results:**

During a median follow up of 7.7 years, 496 individuals (288 men; 58%) developed type 2 diabetes. The median (Q1–Q3) Prx4 level was 0.84 (0.53–1.40) U/l in individuals who developed type 2 diabetes and 0.68 (0.43–1.08) U/l in individuals who did not develop type 2 diabetes. For every doubling of Prx4 levels, the adjusted OR (95% CI) for type 2 diabetes was 1.16 (1.05–1.29) in the whole population; by sex, it was 1.31 (1.14–1.50) for men and 1.03 (0.87–1.21) for women. Further adjustment for other clinical measures did not materially change the results. The addition of Prx4 to a validated diabetes risk score significantly improved the prediction of type 2 diabetes in men (*p* = 0.002 for reclassification improvement).

**Conclusions/interpretation:**

Our findings suggest that elevated serum Prx4 levels are associated with a higher risk of incident type 2 diabetes. For men, taking Prx4 into consideration can improve type 2 diabetes prediction over a validated diabetes risk score; in contrast, there is no improvement in risk prediction for women.

**Electronic supplementary material:**

The online version of this article (doi:10.1007/s00125-014-3278-9) contains peer-reviewed but unedited supplementary material, which is available to authorised users.

## Introduction

Type 2 diabetes mellitus is a leading cause of lifelong morbidity and all-cause mortality in many countries worldwide [1, 2]. The multifactorial and chronic natural history of type 2 diabetes makes it a promising, but challenging, target for therapeutic intervention in its early stages. Evidence suggests that oxidative stress plays a key role in the initiation and progression of type 2 diabetes [3]. Oxidative stress status is characterised as exposure to reactive oxygen or nitrogen species that is not balanced by endogenous antioxidant defences, resulting in increased oxidative damage [4]. A number of studies have identified a relationship between oxidation products or antioxidant levels and the disease state [4–7]. However, clinical or observational data showing associations between oxidative stress markers and type 2 diabetes are scarce [5]. Reasons for the current lack of published population-based studies include technical difficulties in biomarker analysis and an absence of reliable assays [5, 8].

The peroxiredoxin (Prx) antioxidant family comprises six isoforms that have recently been shown to have important functions in cellular antioxidant defence [9]. Experimental studies have shown overexpression of intracellular Prx to occur in animal models of diabetes and obesity [10–12]. Of the six family members, Prx4 is the only isoform encoded by the X chromosome (the human *PRX4* gene is located on Xp22.11) and the only one to be secreted into the circulation [9, 13]. Elevated Prx4 levels appear to protect against diabetes via both local (e.g. intrahepatic or inside pancreatic beta cells) and systemic effects on oxidative stress [9, 13]. Prx4 is stable in the circulation and can be precisely measured using a validated immunoassay [14]. In a recent study, we found circulating Prx4 levels to predict cardiovascular disease risk after accounting for established risk factors [15]. In line with previous studies, we found a cross-sectional association between diabetes and elevated Prx4 levels. We also showed that increased Prx4 levels are associated with some components of the metabolic syndrome (such as hypertension and triglycerols) and with well-established inflammatory markers (such as high sensitivity C-reactive protein [hs-CRP] and procalcitonin) [14–16].

To the best of our knowledge, the potential association between Prx4 and type 2 diabetes risk has not been investigated. Therefore, we aimed to investigate whether circulating Prx4 levels are associated with the development of type 2 diabetes in the general population. Given the sex differences in oxidative stress defence systems and the location of the *PRX4* gene on the X chromosome, we also performed a separate analysis in men and women. We used data from a large population-based cohort study and accounted for potential variations in Prx4 levels over time.

## Methods

### Study population and design

The Prevention of Renal and Vascular End-stage Disease (PREVEND) study includes a Dutch cohort taken from the general population (age range 28–75 years) of Groningen, the Netherlands, between 1997 and 1998. We previously reported details of the study design and of participant recruitment elsewhere (see electronic supplementary material [ESM] Methods for further details) [17, 18]. From the baseline cohort (*n* = 8,592), we first excluded 331 individuals who had diabetes at baseline. These cases were defined by self-reporting of a physician diagnosis or by screening at the first visit (1996–1997). Another 289 participants with no follow-up data or who could not be linked to a pharmacy registry were excluded, leaving 7,972 participants free of baseline diabetes for our cohort analysis. The PREVEND study was approved by the local medical ethics committee of the University Medical Center Groningen and conformed to the principles outlined in the Declaration of Helsinki. All participants gave written informed consent.

### Clinical variables and laboratory markers

All participants attended two outpatient sessions during three rounds of screening between 1997 and 1998 (baseline examination) and 1 January 2007 (third examination). Blood samples were taken after overnight fasting for measuring biomarkers and stored at −80°C. Prx4 in stored serum samples was measured using a novel immunoluminometric assay (interassay CV <20% was 0.51 U/l; intra-assay CV was <8% throughout range of Prx4 levels) [15]. Details on clinical variables and laboratory markers were described previously (see ESM Methods) [15, 19, 20].

### Definition of outcome

Incident type 2 diabetes was defined if one or more of the following criteria were met after baseline recruitment: (1) fasting plasma glucose ≥7.0 mmol/l (126 mg/dl); (2) random sample plasma glucose ≥11.1 mmol/l (200 mg/dl); (3) self-reporting of a physician diagnosis; and (4) initiation of glucose-lowering medication use retrieved from a central pharmacy registry [20].

### Statistical analysis

We performed a logarithmic transformation to normalise the distribution of Prx4. Log base 2 (log_2_) was used to facilitate the interpretation of results per doubling of Prx4 levels. First, we prospectively analysed the association of Prx4 with the risk of new-onset type 2 diabetes using logistic regression in univariate and multivariable-adjusted models. In model 1, we adjusted for age and sex (total population). In model 2, we also adjusted for smoking, family history of diabetes, hypertension and waist circumference. These variables, which are commonly used as predictors of type 2 diabetes risk, are included in the Data from the Epidemiological Study on the Insulin Resistance Syndrome (DESIR) clinical model [21, 22]. Next, we added HDL-cholesterol, triacylglycerols, glucose, insulin resistance (defined as HOMA-IR levels above the sex-specific 75th percentile of HOMA-IR distribution) [19, 23, 24], hs-CRP and 24 h urinary albumin excretion (UAE) in stepwise adjustments to model 2. We also adjusted for all of these clinical and biochemical variables in combination with the variables included in model 2. To investigate potential sex differences in the association between Prx4 level and type 2 diabetes, we calculated the interaction term for Prx4 × sex in model 2, and stratified analyses by sex. Interaction terms were considered statistically significant at *p* < 0.10 [25].

Second, to assess the potential prognostic value of Prx4 for type 2 diabetes risk, we compared the improvement of prediction with the DESIR models, which have been validated in European populations [22, 26]. The DESIR models were chosen because they have separate prediction rules for women and men (see ESM Methods). We calculated the 7.5 year type 2 diabetes risk based on the DESIR models [26] and on a model combining the DESIR models and log_2_Prx4. The following measures were then calculated to assess the improvement in prediction: (1) C-statistic (95% CI), to quantify the discrimination performance of the models (their ability to distinguish between individuals with and without new-onset type 2 diabetes); and (2) integrated discrimination improvement (IDI) and continuous net reclassification improvement (NRI), to examine whether individuals with and without this outcome were correctly reclassified [15, 27, 28].

In secondary analyses, we used data from a second Prx4 measurement to account for changes in Prx4 levels over time. We then fitted fractional polynomials to examine whether the relationship between Prx4 levels and new-onset type 2 diabetes is nonlinear (see ESM Methods). A likelihood ratio test was used to test for nonlinearity (*p* < 0.05).

All statistical analyses were carried out using IBM SPSS (version 19.0; Chicago, IL, USA) and R (version 2.15.2; Vienna, Austria; http://75cran.r-project.org/).

## Results

### Associations of Prx4 with baseline variables and new-onset type 2 diabetes

At baseline, unadjusted median Prx4 levels with interquartile range (Q1–Q3) were 0.71 (0.45–1.16) U/l in men and 0.66 (0.42–1.08) U/l in women (*p* < 0.001). Detailed characteristics of the total population and the corresponding tertiles of serum Prx4 are summarised in Table 1. Across Prx4 tertiles, individuals with higher Prx4 levels were older, more obese, less frequent alcohol drinkers and more likely to have hypertension and higher cholesterol, triglycerols, glucose, hs-CRP, procalcitonin and 24 h UAE. During a median (Q1–Q3) follow up for 7.7 (7.4–8.0) years, 496 individuals (6.2%) developed type 2 diabetes. A comparison of Prx4 levels in individuals who developed new-onset type 2 diabetes vs individuals who remained free of disease is shown in ESM Table 1. Prx4 concentrations were significantly higher in individuals who developed type 2 diabetes than in individuals who did not (0.84 [0.53–1.40] and 0.68 [0.43–1.08] U/l, respectively; *p* < 0.001).

**Table 1:** Baseline clinical and laboratory characteristics of participants

	Total	Sex-specific Prx4 tertiles			
		1st	2nd	3rd	*p* value
No. of participants	7, 972 (100)	2,666 (33.4)	2,604 (32.7)	2,702 (33.9)	**–**
Male	3,909 (49.0)	1,311 (49.2)	1,281 (49.2)	1,352 (50.0)	0.9
Age (years)	48.9 ± 12.5	47.1 ± 11.9	48.6 ± 12.2	51.3 ± 13.1	<0.001
Family history of diabetes	1,566 (19.6)	497 (18.6)	512 (19.7)	557 (20.6)	0.19
Smoking					<0.001
Never	2,340 (29.4)	747 (28)	777 (29.8)	816 (30.2)	
Current	2,738 (34.3)	1,031 (38.7)	896 (34.4)	811 (30.0)	
Former	2,894 (36.3)	888 (33.3)	931 (35.8)	1,075 (39.8)	
Alcohol use					<0.001
≥4 drinks per day	409 (5.1)	115 (4.3)	157 (6.0)	137 (5.1)	
1–3 drinks per day	1,566 (19.6)	587 (22.1)	486 (18.7)	493 (18.2)	
2–7 drinks per week	2,708 (34.0)	968 (36.3)	912 (35.0)	828 (30.6)	
1–4 drinks per month	1,280 (16.1)	409 (15.3)	432 (16.6)	439 (16.2)	
Almost never	2,009 (25.2)	587 (22.1)	617 (23.7)	805 (30.0)	
Systolic BP (mmHg)	124.0 ± 19.3	121.2 ± 17.6	123.5 ± 18.9	127.1 ± 20.9	<0.001
Diastolic BP (mmHg)	71.7 ± 9.7	70.4 ± 9.1	71.7 ± 9.8	72.8 ± 10.1	<0.001
Hypertension	2,238 (28.1)	576 (21.6)	706 (27.1)	956 (35.4)	<0.001
BMI (kg/m^2^)	26.0 ± 4.2	25.4 ± 3.8	26.0 ± 4.1	26.7 ± 4.5	<0.001
Waist circumference (cm)	88.1 ± 12.9	86.4 ± 12.1	87.8 ± 12.6	90.2 ± 13.6	<0.001
Glucose (mmol/l)	4.7 ± 0.6	4.7 ± 0.6	4.7 ± 0.6	4.8 ± 0.7	<0.001
Insulin (pmol/l)	47.4 (33–70.8)	43.8 (31.2–69.4)	46.8 (32.4–69.6)	53.4 (36–82.2)	<0.001
HOMA-IR	1.63 (1.10–2.55)	1.48 (1.03–2.20)	1.61 (1.07–2.47)	1.84 (1.22–3.06)	<0.001
Total cholesterol (mmol/l)	5.64 ± 1.12	5.61 ± 1.08	5.62 ± 1.14	5.68 ± 1.14	0.04
HDL cholesterol (mmol/)	1.33 ± 0.40	1.37 ± 0.40	1.33 ± 0.39	1.29 ± 0.4	<0.001
Triacylglycerol (mmol/l)	1.15 (0.84–1.66)	1.12 (0.81–1.56)	1.11 (0.82–2.38)	1.22 (0.88–1.8)	<0.001
hs-CRP (mg/l)	1.26 (0.55–2.88)	0.94 (0.42–2.09)	1.21 (0.56–2.73)	1.78 (0.78–3.70)	<0.001
Procalcitonin (ng/ml)	0.016 (0.013–0.020)	0.015(0.013–0.019)	0.016 (0.013–0.019)	0.016 (0.013–0.021)	<0.001
UAE (mg/24 h)	9.3 (6.3–16.8)	8.7 (6.2–14.2)	9.1 (6.2–15.6)	10.3 (6.4–22.0)	<0.001

Data are mean (±SD) and median (quartiles 1 and 3) for continuous variables and *n* (%) for categorical variables in the total population and corresponding to sex-specific Prx4 tertiles

*p* values from univariate analyses (for comparison across Prx4 tertiles) were determined using ANOVA or Kruskal–Wallis for continuous variables or *χ* ^2^ tests for categorical variables

### Primary analysis of risk

ORs (95% CI) of univariate and multivariable-adjusted models for the risk of new-onset type 2 diabetes for the total population and separately for men and women are shown in Table 2. In the whole population, the age- and sex-adjusted OR was 1.55 (1.21–1.99) for type 2 diabetes when comparing the top tertile to the bottom tertile of Prx4 (*p* for trend <0.001). In clinical model 2, adjusted for diabetes risk factors, the association of Prx4 with type 2 diabetes was attenuated, with an OR of 1.24 (0.96–1.60). The corresponding OR per unit increase in log_2_Prx4 (i.e. per doubling of Prx4 levels) was 1.16 (1.05–1.29). The direction and strength of the relationship between log_2_Prx4 and type 2 diabetes was similar when we refitted the clinical model and either included BMI instead of waist circumference (OR 1.16 [1.04–1.29]) or added both waist circumference and BMI to the same model (OR 1.16 [1.04–1.28]). Separate adjustments for HDL-cholesterol (OR 1.14 [1.02–1.27]), triglycerols (OR 1.12 [1.00–1.24]), glucose (OR 1.16 [1.03–1.29]), insulin resistance (OR 1.11 [1.00–1.24]), hs-CRP (OR 1.12 [1.00–1.25]) and UAE (OR 1.15 [1.04–1.28]) combined with the DESIR clinical model did not materially change the association between Prx4 and type 2 diabetes. When we adjusted for all of these variables in combination with the DESIR clinical model, the OR was 1.06 (0.93–1.18).

**Table 2:** Association of Prx4 with new-onset type 2 diabetes

Analysis by group	Prx4 tertile, U/l			OR (95% CI) per log_2_ unit increase	*p* value
	1st	2nd	3rd		
Total (*n* = 7,952)					
No. of cases (%)	115 (4.4)	156 (6.0)	225 (8.2)	–	–
Crude analysis	1.00	1.39 (1.05–1.81)	1.87 (1.45–2.42)	1.37 (1.24–1.52)	<0.001
Model 1	1.00	1.29 (1.00–1.59)	1.55 (1.21–1.99)	1.27 (1.15–1.41)	<0.001
Model 2	1.00	1.20 (0.92–1.56)	1.24 (0.96–1.60)	1.16 (1.05–1.29)	0.005
Men (*n* = 3,909)					
No. of cases (%)	63 (4.8)	85 (6.6)	140 (10.6)	–	–
Crude analysis	1.00	1.30 (0.90–1.88)	2.32 (1.67–3.27)	1.46 (1.28–1.67)	<0.001
Model 1	1.00	1.21 (0.84–1.74)	1.94 (1.40–2.73)	1.37 (1.20–1.57)	<0.001
Model 2	1.00	1.14 (0.79–1.64)	1.69 (1.21–2.38)	1.31 (1.14–1.50)	<0.001
Women (*n* = 4,063)					
No. of cases (%)	55 (4.0)	66 (5.0)	87 (6.3)	–	–
Crude analysis	1.00	1.03 (0.69–1.53)	1.40 (0.97–2.04)	1.27 (1.07–1.48)	0.004
Model 1	1.00	1.06 (0.72–1.56)	1.28 (0.89–1.85)	1.19 (1.01–1.39)	0.03
Model 2	1.00	1.00 (0.67–1.47)	0.98 (0.67–1.40)	1.03 (0.87–1.21)	0.70

Model 1 is adjusted for age and sex (total population); model 2 is adjusted for the covariates in model 1 and smoking, waist circumference, hypertension and family history of diabetes

Given the sex-related differences in Prx4 concentration and diabetes risk factors, we next stratified the analysis by sex. In men, age-adjusted and multivariable-adjusted (for the variables in model 2) ORs for type 2 diabetes were 1.37 (1.20–1.57) and 1.31 (1.14–1.50), respectively, per doubling of Prx4 levels. When we further adjusted for HDL-cholesterol, triglycerols, hs-CRP, UAE, insulin resistance and glucose in combination with the DESIR clinical model, the OR was 1.22 (1.05–1.40). In women, the age-adjusted OR for type 2 diabetes was 1.19 (1.01–1.39) per doubling of Prx4 levels. After adjusting for the variables in model 2, the association was attenuated to non-significance (OR 1.03 [0.87–1.21]; *p* = 0.70). For the interaction term between Prx4 and sex, there was a trend toward significance (*p* = 0.13).

In the whole population, the DESIR clinical and clinical–biological models had C-statistics (95% CI) of 0.754 (0.734–0.773) and 0.819 (0.799–0.840), respectively, for new-onset type 2 diabetes risk. The addition of Prx4 to the clinical model modestly improved the C-statistic to 0.758 (0.734–0.773; *p* = 0.02), and led to an IDI of 0.0012 (*p* = 0.09) and a continuous NRI of 0.14 (*p* = 0.002). The addition of Prx4 to the clinical–biological model modestly improved the C-statistic to 0.823 (0.802–0.843; *p* = 0.03), and led to an IDI of 0.0013 (*p* = 0.2) and a continuous NRI of 0.15 (*p* = 0.002). In men, the addition of Prx4 to the DESIR clinical model significantly improved the C-statistic from 0.701 to 0.710 (*p* = 0.04), and improved both the IDI (0.003; *p* < 0.01) and the continuous NRI (0.21; *p* < 0.001). The DESIR clinical model with the addition of glucose had a C-statistic of 0.831 (0.807–0.856). The addition of Prx4 to the DESIR clinical model with glucose minimally improved the C-statistic (change of +0.003; *p* = 0.33), but led to an IDI of 0.003 (*p* = 0.04) and a continuous NRI of 0.15 (*p* = 0.01) in men. The addition of Prx4 to the DESIR clinical–biological model, as a second reference (showing a C-statistic of 0.831), minimally improved the C-statistic (change of +0.002; *p* = 0.37), but led to an IDI of 0.0031 (*p* = 0.04) and a continuous NRI of 0.15 (*p* = 0.01). In women, the addition of Prx4 to the DESIR clinical model did not improve the prediction in terms of discrimination (the C-statistic changed from 0.823 to 0.824; *p* = 0.31) and reclassification (IDI of 0.0001, *p* = 0.82; continuous NRI of 0.034, *p* = 0.63).

### Secondary analysis of risk

**Fig. 1:**
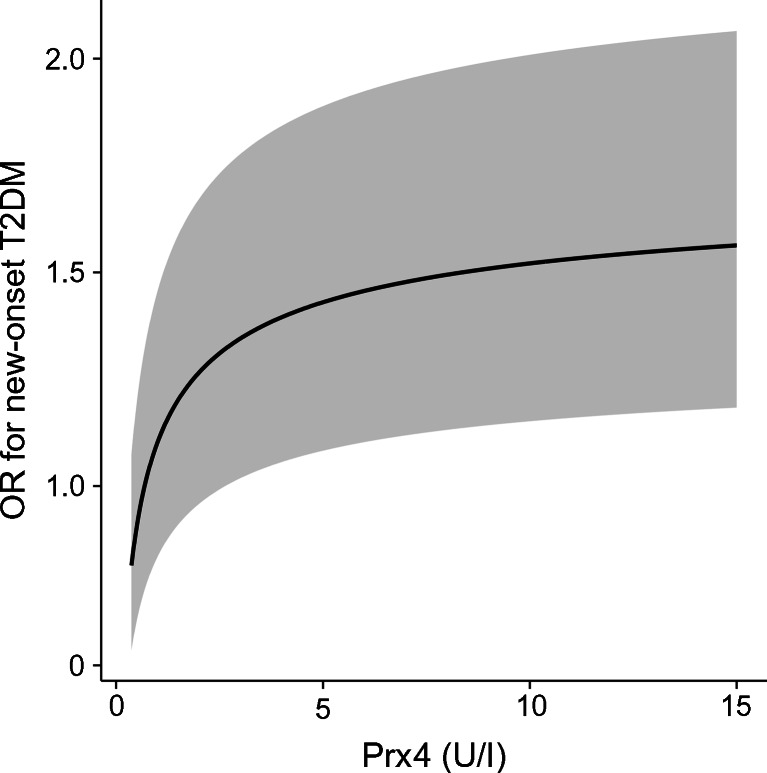
Nonlinear relationship between Prx4 and new-onset type 2 diabetes mellitus. Data are shown for 7,952 participants without diabetes at baseline. A fractional polynomials model was fitted to examine the linearity and determine the functional form of Prx4. The optimal transformation of Prx4 was one in which the term Prx4^−0.5^ was incorporated; OR for diabetes = exp(β × (*x*-median)^−0.5^), where β is the regression coefficient for transformed Prx4 (*x*). The plotted ORs (solid line) and 95% CI (shaded area) were calculated for different values of Prx4 compared with the median Prx4 value, which was used as the reference. Prx, peroxiredoxin; T2DM, type 2 diabetes mellitus

Fractional polynomials showed that the relationship between Prx4 and new-onset type 2 diabetes deviated from linearity (*p* for nonlinearity <0.0001). The best-fit model included the power −0.5 for Prx4 (*p* for nonlinear function <0.0001). The model was adjusted for the DESIR clinical model. Fig. 1 depicts the nonlinear relationship between Prx4 and type 2 diabetes. The solid line indicates the ORs (95% CIs) [26] plotted against the functional form of Prx4. To estimate the risk of type 2 diabetes at different values of Prx4, we selected the median Prx4 value as a reference. Accordingly, the risk of type 2 diabetes increases steeply within the lower range of Prx4 until a plateau is reached at the highest values of Prx4. Finally, we accounted for the variation in Prx4 concentrations over time in the PREVEND study. Prx4 levels were 0.55 (0.37–0.84) U/l in men and 0.48 (0.37–0.74) U/l in women at the third examination. The β-coefficient for regression of the second measurement of Prx4 on the baseline values of Prx4 was 0.527 for men and 0.491 for women. After adjusting for regression dilution bias, the OR per single unit increase in log_2_Prx4 was 1.67 (1.28–2.17) and 1.06 (0.76–1.48) for men and for women, respectively, in model 2.

## Discussion

In this large prospective cohort with no diabetes at baseline, we demonstrated that circulating Prx4 concentrations are positively associated with an increased risk of new-onset type 2 diabetes even after adjusting for established diabetes risk factors. The addition of Prx4 to a validated diabetes risk score significantly improved risk prediction for new-onset type 2 diabetes in terms of discrimination and reclassification. The positive relationship between Prx4 and type 2 diabetes was nonlinear (i.e. curved) and statistically significant for men.

The strengths of our epidemiological study are its large sample size, prospective design and verification of new-onset type 2 diabetes cases. We used a reliable assay to measure circulating Prx4 in samples obtained at both baseline and the third examination. The repeated Prx4 measurement enabled us to account for potential variations over time or for measurement error using the regression dilution ratio. Regression dilution bias was described as attenuation in the estimated effect of exposure to a risk factor (e.g. Prx4) to disease risk (e.g. type 2 diabetes) when a single measurement was used [29]. Nevertheless, some limitations of our study should be stated. The PREVEND cohort predominantly comprises white adults, and it is therefore unclear whether our findings can be generalised to nonwhite populations. By design, our cohort was enriched for individuals with urine albumin concentrations above 10 mg/l at baseline. However, a weighted method performed to compensate for this did not affect the results. Nevertheless, we also investigated the adjusted OR for 24 h UAE as a potential confounding factor. Similar to most observational studies, our cohort was not originally set up to investigate diabetes. Since individuals with type 2 diabetes can remain undiagnosed for several months to years, we might therefore have missed false-negative cases in the remainder of the cohort [22]. However, this would weaken the association rather than generating a false-positive association. Nonetheless, the incidence of type 2 diabetes in the PREVEND cohort is similar to current estimates of diabetes in European adults [2]. The study is an observational investigation; therefore, causal relationships between Prx4 as an antioxidant biomarker and type 2 diabetes cannot be inferred. In addition, although we accounted for confounding by established diabetes risk factors, the potential for unobserved confounding remains. Finally, we used logistic regression in our cohort study because disease-associated changes have been detected at regular screening visits or shortly thereafter in the PREVEND study. Thus, estimated survival and hazards cannot be accurately calculated using this type of follow up [22]. However, we and others have shown that survival models do not necessarily perform better than logistic ones [22, 30].

There is accumulating evidence that the thiol-dependent antioxidant family member, Prx4, plays a key role in oxidant scavenging and in signalling cascades that protect against oxidative damage [9, 11, 31]. In an experimental study of an animal model of type 1 diabetes, transgenic mice overexpressing human Prx4 had significantly higher Prx4 expression in pancreatic islets and reduced hyperglycaemia compared with wild-type mice [11]. In other words, Prx4 supplementation in vivo has a protective effect against diabetes and can lead to improved insulin resistance. Consistent with this, increased *Prx4* gene expression has been observed after high-fat diet-induced beta cell dysfunction in mice [32]. Increased reactive oxygen species production is a key change in the development of insulin resistance and in early stage beta cell dysfunction in both human patients and animal models of type 2 diabetes [32, 33]. Changes in Prx4 expression regulate the cellular redox state, and suggest that oxidative metabolism is enhanced in the islets of animals receiving high-fat or high-carbohydrate diets [32]. Prx4 may also have a pivotal role in the suppression of apoptosis and in progenitor cell proliferation in vivo to protect against oxidative stress-induced beta cell dysfunction [11].

Moreover, Prx4 is the only Prx family member known to be secreted [9, 11, 13, 34]. Cross-sectional in vivo studies in humans also reported elevated Prx4 in type 2 diabetes patients [9, 15, 31]. El Eter et al reported significantly higher Prx4 levels in type 2 diabetes patients with peripheral atherosclerotic disease than in healthy controls [31]. Nabeshima et al also found higher serum Prx4 levels in male patients with type 2 diabetes than in a group of healthy males [9]. A recent analysis of clinical data showed increased serum Prx4 levels in septic patients compared with healthy individuals [16, 35]. In line with the latter study, we previously reported a positive association between Prx4 levels and inflammatory markers (such as hs-CRP), measures of adiposity (such as BMI), BP and glucose [15]. These factors underlie the central biological pathways of metabolic syndrome and type 2 diabetes [15, 36, 37]. Prx4 may protect against the metabolic abnormalities leading to type 2 diabetes so that upregulated intracellular Prx4 synthesis and augmented extracellular Prx4 levels suppress oxidative stress and ameliorate local (e.g. hepatic or islet cells) and systemic inflammatory signalling and insulin sensitivity [9, 38]. Prx4 promotes antioxidant activity via several pathways, such as nuclear factor-κB (NF-κB) [39], p53 [40], thromboxane A2 receptor [41] and NF-E2-related factor 2 (Nrf2) [15, 42]. A recent clinical trial showed that treatment with the Nrf2 antagonist, a specific antioxidant that affects the Prx4 pathway, is an effective intervention against the decline in renal function in patients with chronic kidney disease and type 1 diabetes [15, 43].

In our study, we extended these in vitro and in vivo experimental studies by investigating the relationship between Prx4 and type 2 diabetes. We prospectively examined whether Prx4 has an additive effect on type 2 diabetes prediction. First, we estimated type 2 diabetes risk in our population using a validated diabetes risk score. These estimates were then used to evaluate the predictive value of Prx4 when added to the DESIR models. In the total population, the addition of Prx4 to the DESIR clinical model statistically improved disease prediction in terms of discrimination and reclassification. Given the sex differences in Prx4 levels and diabetes risk factors, we next stratified the analysis by sex. We observed that Prx4 predicted the risk of new-onset type 2 diabetes independently of established diabetes risk factors only in men. In women, the addition of Prx4 did not improve risk prediction for type 2 diabetes. The reason that the association between Prx4 and type 2 diabetes is stronger in men than in women is unknown. The *PRX4* gene is located on the X chromosome; therefore, further studies are necessary to investigate whether potential differences in gene expression or in sex hormones contributes to differences between men and women in the Prx4 response to oxidative stress. Finally, our findings require validation to confirm the utility of Prx4 in type 2 diabetes risk prediction.

In conclusion, our results suggest that elevated circulating Prx4 is associated with an increased risk of type 2 diabetes, even after adjusting for diabetes risk factors, in a population-based cohort study. Prx4 was more strongly associated with type 2 diabetes risk in men than in women. In men, Prx4 analysis can improve the prediction of type 2 diabetes above that of a validated diabetes risk score; in contrast, Prx4 showed no added predictive value in women. Further studies are warranted to elucidate the underlying mechanisms of action.

## Electronic supplementary material

Below is the link to the electronic supplementary material.

ESM Methods (PDF 91 kb)

ESM Table 1 (PDF 79 kb)

## Abbreviations

DESIR: Data from the Epidemiological Study on the Insulin Resistance Syndrome
Hs-CRP: High sensitivity C-reactive protein
IDI: Integrated discrimination improvement
NRI: Net reclassification improvement
PREVEND: Prevention of Renal and Vascular End-stage Disease
Prx: Peroxiredoxin
UAE: Urinary albumin excretion
