# Supplementary material for: Circulating peroxiredoxin 4 and type 2 diabetes risk: the Prevention of Renal and Vascular Endstage Disease (PREVEND) study
Source: Diabetologia. 2014 Jun 4;57(9):1842–9. doi: 10.1007/s00125-014-3278-9 (PMC4119240; doi:10.1007/s00125-014-3278-9)
Supplement: Supplementary file 1 — (PDF 91 kb) [file 125_2014_3278_MOESM1_ESM.pdf]

Supplementary text

Methods

Study population and design

The Prevention of Renal and Vascular End-stage Disease (PREVEND) study is a Dutch cohort drawn from the general population (age ranged between 28 and 75 years) of the city of Groningen, the Netherlands between 1997 and 1998. We have reported details of the study design and recruitment of participants elsewhere [1, 2]. In brief, 40,856 individuals (47.8%) completed a questionnaire on demographics, history of cardiovascular disease and metabolic traits, medication use and pregnancy prior to their first visit and collected early morning urine sample in a vial to measure urinary albumin concentration (UAC). After exclusion of individuals who were unable or unwilling to participate, individuals using insulin and pregnant women, the baseline PREVEND study consisted of a total of 6,000 individuals with UAC ≥ 10 mg/L and a random sample of individuals with UAC < 10 mg/l (n=2,592), together resulting in a baseline cohort of 8,592.

Measurement of clinical variables and laboratory markers

All participants underwent two outpatient visits during three rounds of screening from 1997-1998 (baseline examination) until January 1st 2007 (third examination). In each round, the participants were assessed for demographics, anthropometric measurements, cardiovascular and metabolic risk factors, health behaviours, and medical family history and to collect two 24-hour urine samples on two consecutive days. Furthermore, information on medication use was substantiated with use of pharmacy-based data from all community pharmacies in the city of Groningen[3]. Smoking and alcohol use were based on self-reports. We defined hypertension based on self-report of diagnosis by a physician, measured hypertension (≥140/90 mmHg systolic/diastolic blood pressure) or the use of blood pressure-lowering agents. Metabolic syndrome was defined according to the National Cholesterol Education Program's Adult Treatment Panel III report (ATP III) criteria[4]. Insulin resistance was assessed based on HOMA-IR that is calculated by the following formula: [glucose (mmol/l) × insulin (mU/l]/22.5 [5]. We defined insulin resistance as a HOMA-IR score in upper sex-specific quartiles (≥2.81 in men and ≥2.31 in women) [6]. In all participants, blood samples for measurements of biomarkers were taken after an overnight fast and stored at -80°C until assessment of biomarkers. The assays were performed in EDTA-plasma aliquots without previous thawing and refreezing. Insulin was measured with an AxSym autoanalyzer (Abbott Diagnostics, Amstelveen, The Netherlands). HDL cholesterol was measured with a homogeneous method (direct HDL, Aeroset System; Abbott Laboratories, Abbott Park, IL). Triglycerides were measured enzymatically. High-sensitivity C-reactive protein (hs-CRP) was determined by nephelometry (BN II; Dade Behring, Marburg, Germany). Urinary albumin excretion was measured as the mean of two 24-h urine collections by nephelometry with a threshold of 2.3 mg/liter (Dade Behring Diagnostic, Marburg,

Germany). Procalcitonin was measured by a novel commercially available immunoluminometric assay (BRAHMS PCT sensitive LIA; Hennigsdorf, Germany)[6]. 24-hour urinary albumin excretion (UAE) − given as the mean of the two 24-hour urine excretions − was measured by nephelometry with a threshold of 2.3 mg/L and intra- and inter-assay coefficients of variation of less than 2.2% and less than 2.6%, respectively (Dade Behring Diagnostic, Marburg, Germany). All technicians were blinded to the participants' characteristics[6].

Statistical analysis

Data on a second Prx4 measurement were available at the third examination. We used these data to account for changes in Prx4 levels over time. To estimate regression dilution ratios, we calculated the regression coefficient (called the reliability coefficient, λ) by regression of second measurement of Prx4 on the baseline values of Prx4[7]. Using these data, the estimated odds ratio corrected for regression dilution bias can then be calculated by the following formula:

$$\text{Estimated risk} = \exp\left(\ln(OR_{Prx4\text{-}T2DM}) \times \lambda^{-1}\right).$$

We fitted fractional polynomials to examine the relationship of Prx4 levels with new-onset type 2 diabetes.

A weighted method was performed to compensate the baseline enrichment for the PREVEND participants with urinary albumin concentration (UAC) ≥10 mg/l. Given the frequency of individuals with UAC ≥10 mg/L (24.4%) in our general population [1, 2], we calculated the weight by sampling fractions. Those with UAC ≥10 mg/l had weight equal to 0.35 and those with UAC <10 mg/l weight equal to 2.51. For most baseline variables, <1% was missing; however, this was up to 8% for self-reported variables. We performed a single imputation with predictive mean matching for missing data. This method can be used for skewed data with less than 10% missingness, because it produces less biased estimates for non-linear models and imputations remain in the metric of the observed data [8, 9].

Detailed description of the existing models to which Prx4 was added as a predictor of type 2 diabetes.

1- The DESIR clinical model [10]

The absolute risk of type 2 diabetes is calculated as:
exp(linear predictor)/(1 + exp(linear predictor))

where, linear predictor (if men)= -10.45 + 0.72 (if current smoker, else 0) + 0.081 × waist circumference (cm) + 0.50 (if hypertension, else 0)
linear predictor (if women)= -11.81 + 1.09 (if family history of diabetes, else 0) + 0.095 × waist circumference (cm) + 0.64 (if hypertension, else 0);

2- The DESIR clinical-biological model

The absolute risk of type 2 diabetes is calculated as:
exp(linear predictor)/(1 + exp(linear predictor))

where, linear predictor (if men)= -10.53 + 0.88 (if current smoker, else 0) + 0.06× waist circumference (cm) + ln(glucose/mean of glucose) ×10.15)+ ((ln(glucose/mean of glucose)^2) ×24.16)+(ln(GGT)×0.39).
linear predictor (if women) = -18.91 + 0.8 (if family history of diabetes, else 0) + 4.38 × (ln(BMI))+ (ln(glucose/mean of glucose) ×9.66)+ ((ln(glucose/mean of glucose)^2) × 23.89) + (ln(TG) × 0.95).

References

[1] Lambers Heerspink HJ, Brantsma AH, de Zeeuw D, Bakker SJ, de Jong PE, Gansevoort RT (2008) Albuminuria assessed from first-morning-void urine samples versus 24-hour urine collections as a predictor of cardiovascular morbidity and mortality. Am J Epidemiol 168: 897-905

[2] Mahmoodi BK, Gansevoort RT, Veeger NJ, et al. (2009) Microalbuminuria and risk of venous thromboembolism. JAMA : the journal of the American Medical Association 301: 1790-1797

[3] Monster TB, Janssen WM, de Jong PE, de Jong-van den Berg LT, REnal PSGPo, Vascular ENTSD (2002) Pharmacy data in epidemiological studies: an easy to obtain and reliable tool. Pharmacoepidemiology and drug safety 11: 379-384

[4] Third Report of the National Cholesterol Education Program (NCEP) Expert Panel on Detection E, and Treatment of High Blood Cholesterol in Adults (Adult Treatment Panel III) final report. (2002) National Cholesterol Education Program (NCEP) Expert Panel on Detection, Evaluation, and Treatment of High Blood Cholesterol in Adults (Adult Treatment Panel III). Circulation 106: 3143-3421

[5] Matthews DR, Hosker JP, Rudenski AS, Naylor BA, Treacher DF, Turner RC (1985) Homeostasis model assessment: insulin resistance and beta-cell function from fasting plasma glucose and insulin concentrations in man. Diabetologia 28: 412-419

[6] Abbasi A, Corpeleijn E, Postmus D, et al. (2010) Plasma procalcitonin is associated with obesity, insulin resistance, and the metabolic syndrome. J Clin Endocrinol Metab 95: E26-31

[7] Knuiman MW, Divitini ML, Buzas JS, Fitzgerald PE (1998) Adjustment for regression dilution in epidemiological regression analyses. Ann Epidemiol 8: 56-63

[8] Marshall A, Altman DG, Holder RL (2010) Comparison of imputation methods for handling missing covariate data when fitting a Cox proportional hazards model: a resampling study. BMC Med Res Methodol 10: 112

[9] van Buuren S, Boshuizen HC, Knook DL (1999) Multiple imputation of missing blood pressure covariates in survival analysis. Statistics in medicine 18: 681-694

[10] Balkau B, Lange C, Fezeu L, et al. (2008) Predicting diabetes: clinical, biological, and genetic approaches: data from the Epidemiological Study on the Insulin Resistance Syndrome (DESIR). Diabetes care 31: 2056-2061
