# Supplementary material for: Circulating peroxiredoxin 4 and type 2 diabetes risk: the Prevention of Renal and Vascular Endstage Disease (PREVEND) study
Source: Diabetologia. 2014 Jun 4;57(9):1842–9. doi: 10.1007/s00125-014-3278-9 (PMC4119240; doi:10.1007/s00125-014-3278-9)
Supplement: Supplementary file 2 — (PDF 79 kb) [file 125_2014_3278_MOESM2_ESM.pdf]

**ESM Table 1.** Baseline characteristics of non-converters and those who developed new-onset type 2 diabetes			
	**Non-converters**	**Incident cases of type 2 diabetes**	***p* value**
No. of participants	7,476 (93.8)	496 (6.2)	-
Male	3, 621 (48.4)	288 (58.1)	<0.001
Age (years)	48.5 ± 12.5	56.5 ± 10.7	<0.001
Family history of diabetes	1, 391 (18.6)	175 (35.3)	<0.001
Smoking			
Current	2566 (34.3)	172 (34.7)	
Former	2692 (36.0)	202 (40.7)	0. 03
Never	2218 (29.7)	122 (24.6)	
Alcohol use			
>= 4 drinks per day	383 (5.1)	26 (5.2)	
1-3 drinks per day	1476 (19.7)	90 (18.1)	
2-7 drinks per week	2568 (34.3)	140 (28.2)	0.01
1-4 drinks per month	1194 (16.1)	86 (17.3)	
Almost never	1855 (24.8)	154 (31.0)	
Systolic blood pressure (mmHg)	122.9 ± 19.0	135.4 ± 20.5	<0.001
Diastolic blood pressure (mmHg)	71.3 ± 9.6	76.2 ± 9.5	<0.001
Hypertension	1593 (24.8)	197 (50.0)	<0.001
BMI (kg/m^2)	25.7 ± 4.0	29.5 ± 4.8	<0.001
Waist circumference (cm)	87.2 ± 12.7	98.7 ± 12.3	<0.001
Glucose (mmol/l)	4.7 ±0.6	5.6 ± 0.8	<0.001
Insulin (pmol/l)	45 (32.4-65.4)	76.8 (51.6-118.8)	<0.001
HOMA-IR	1.55 (1.06-2.33)	3.17 (2.08-5.19)	<0.001
Total cholesterol (mmol/l)	5.62 ± 1.12	6.01 ± 1.13	<0.001
HDL cholesterol (mmol/l)	1.35 ± 0.40	1.11 ± 0.29	<0.001
Triglycerides (mmol/l)	1.12 (0.81-1.57)	1.66 (1.21-2.38)	<0.001
hs-CRP (mg/l)	1.20 (0.54-2.78)	2.25 (1.16-4.47)	<0.001
Procalcitonin (ng/ml)	0.016 (0.013-0.019)	0.018 (0.015-0.022)	<0.001
Peroxiredoxin 4 (U/l)	0.678 (0.430-1.080)	0.839 (0.534-1.400)	<0.001
UAE (mg/24hour)	9.0 (6.2-16.0)	15.3 (8.3-37.3)	<0.001

Data are *n*, mean ± SD, median (Q1-Q3) for continuous variables or *n* (%) for categorical variables, presented non-adjusted values.
P values from univariate analyses (for the comparison between non-converters and those who developed type 2 diabetes were determined using *t* test or the Mann-Whitney *U* test for continuous variables or χ^2 test for categorical variables,
BMI is the weight in kilogram divided by the square of the height in meters, UAE, urine albumin excretion and hs-CRP, high sensitivity C-reactive protein and HOMA-IR, homeostatic model assessment-insulin resistance.
